# Resistance Modulation Action, Time-Kill Kinetics Assay, and Inhibition of Biofilm Formation Effects of Plumbagin from *Plumbago zeylanica* Linn

**DOI:** 10.1155/2019/1250645

**Published:** 2019-11-26

**Authors:** Emmanuel B. A. Adusei, Reimmel K. Adosraku, James Oppong-Kyekyeku, Cedric D. K. Amengor, Yakubu Jibira

**Affiliations:** ^1^Department of Pharmaceutical Chemistry, Faculty of Pharmacy and Pharmaceutical Sciences, College of Health Sciences, Kwame Nkrumah University of Science and Technology, Kumasi, Ghana; ^2^Department of Pharmaceutical Chemistry, School of Pharmacy, University of Health and Allied Sciences, Ho, Ghana; ^3^Department of Pharmacology, Faculty of Pharmacy and Pharmaceutical Sciences, College of Health Sciences, Kwame Nkrumah University of Science and Technology, Kumasi, Ghana

## Abstract

Antimicrobial resistance (AMR) is a threat to the prevention and treatment of the increasing range of infectious diseases. There is therefore the need for renewed efforts into antimicrobial discovery and development to combat the menace. The antimicrobial activity of plumbagin isolated from roots of *Plumbago zeylanica* against selected organisms was evaluated for resistance modulation antimicrobial assay, time-kill kinetics assay, and inhibition of biofilm formation. The minimum inhibitory concentrations (MICs) of plumbagin and standard drugs were determined via the broth microdilution method to be 0.5 to 8 *μ*g/mL and 0.25–128 *μ*g/mL, respectively. In the resistance modulation study, MICs of the standard drugs were redetermined in the presence of subinhibitory concentration of plumbagin (4 *μ*g/mL), and plumbagin was found to either potentiate or reduce the activities of these standard drugs with the highest potentiation recorded up to 12-folds for ketoconazole against *Candida albicans*. Plumbagin was found to be bacteriostatic and fungistatic from the time-kill kinetics study. Plumbagin demonstrated strong inhibition of biofilm formation activity at concentrations of 128, 64, and 32 *μ*g/mL against the test microorganisms compared with ciprofloxacin. Plumbagin has been proved through this study to be a suitable lead compound in antimicrobial resistance drug development.

## 1. Introduction

Occurrence of infectious diseases in recent times is attributed mostly to bacteria; a result of prominent resistance strains of organisms to available antibiotics. These strains include drug-resistant *Shigella*, multidrug-resistant tuberculosis (MDR-TB), multidrug-resistant *Escherichia coli* and *Neisseria gonorrheae*, methicillin-resistant *Staphylococcus aureus*, and extensively resistant Gram-negative organisms like *Pseudomonas aeruginosa* and *Acinetobacter baumannii* [1]. Antimicrobial resistance stems from multifactorial causes which include irrational drug use, failure to complete prescribed dosage, prolonged drug use, and therapy duration [2, 3]. Infections become difficult to treat due to AMR, while surgical and other medical procedures become high-risk intervention causing disability, prolonged sickness, and death [4]. Other consequences of AMR include passage of antibiotic resistant diseases to others and increase in economic burden on families, societies, and healthcare systems [4]. Modulation of the activities of commercial antimicrobials by phytochemicals may reverse the mechanism of resistance developed by the organisms [5]. Synergism of conventional antibiotics is being researched into in recent times to establish possible candidates for the discovery and development of new drugs to tackle the menace of antimicrobial resistance [6].

Time-kill kinetics assays help understand interactions that exist between microbial strains and antimicrobial agents. The assay shows a concentration or time-dependent test effect of antimicrobial agents on strains of microorganisms. It determines antimicrobial agents as bacteriostatic/fungistatic or bactericidal/fungicidal [7]. Bacteria develop resistance to drugs in different ways including formation of biofilms, active efflux of drugs, drug inactivation caused by enzyme secretion, and drug target site alteration [5]. Biofilm formation reduces the penetrating abilities, and as such bacteria producing biofilms are not affected by the mode of action of antibiotics [8]. Biofilms refer to cells of microbes embedded in self-produced matrix of extrapolymeric substances attached irreversibly to a surface. Biofilms constitute 65% of microbial infections, and bacteria living in them develop resistance to antimicrobial agents a thousand times than those existing in free-living forms (planktonic forms) [9]. Potential agents to be considered in antimicrobial resistant drug development should therefore demonstrate strong biofilm reduction or inhibition activity [9].

*Plumbago zeylanica* Linn (Plumbaginaceae) is a bushy and straggling perennial shrub that grows between 1.5–3.0 m in height. It is distributed throughout tropical Africa with collection of the plant reported in Senegal, Guinea, Sierra Leone, Liberia, Ghana, Togo, Nigeria, Zambia, and Equatorial Guinea. Its roots are used traditionally to treat tuberculosis, gonorrhoea, diarrhoea, syphilis, wounds, and rheumatic pains and swellings [10, 11]. The major component of the roots of the plant is plumbagin (5-hydroxy-2-methyl-1,4-naphthoquinone), a yellowish pigment also found naturally in plants of the Droseraceae, Ebenaceae, Iridaceae, Ancestrocladaceae, Drosophyllaceae, and Nepenthaceae families [12]. It has been reported to possess several biological activities including anticancer, antioxidant, antibacterial, antifungal, anti-inflammatory, antimalarial, and antiparasitic [13–19]. Plumbagin isolated from *Plumbago scandens* was evaluated for antibacterial activity by Paiva et al. using the broth macrodilution method. It exhibited a minimum inhibitory concentration (MIC) of 1.56 *μ*g/mL against *Staphylococcus aureus* [18].

The resistance modulation action, time-kill kinetics, and effects on biofilm formation of plumbagin isolated from *Plumbago zeylanica* plant were investigated in this study and are hereby reported as our findings.

## 2. Materials and Methods

### 2.1. Materials and Reagents

The reagents used included the following: ethyl acetate (BDH Chemicals, UK), petroleum ether [VWR chemicals, U.S.A], acetic acid (Needham Market Suffolk, UK), dimethyl sulfoxide (DMSO) [Sigma-Aldrich, U.S.A], 3-(4, 5-dimethylthiazol-2-yl)-2, 5 diphenyl tetrazolium bromide (MTT), silica gel (70 : 230 mesh size) [Merck, U.S.A], silica gel coated TLC plates [Merck, Germany], crystal violet, 70% v/v ethanol [Fischer Scientific, U.K], ciprofloxacin [Sigma-Aldrich, U.S.A], ketoconazole [Sigma-Aldrich, U.S.A], amoxicillin [Sigma-Aldrich, U.S.A], and ampicillin [Sigma-Aldrich, U.S.A].

### 2.2. Test Organisms

Pure cultures of *Staphylococcus aureus* (ATCC 25923), *Escherichia coli* (ATCC 25922), *Pseudomonas aeruginosa* (ATCC 27853), *Klebsiella pneumoniae* (ATCC 10031), and *Candida albicans* (ATCC 10231) were obtained from the Microbiology laboratory of the Department of Pharmaceutics, Faculty of Pharmacy and Pharmaceutical Sciences, Kwame Nkrumah University of Science and Technology (KNUST), Kumasi, Ghana.

### 2.3. Collection of Plant Material

The *Plumbago zeylanica* roots were collected from the Physic garden in the Faculty of Pharmacy and Pharmaceutical Sciences, Kwame Nkrumah University of Science, and Technology (KNUST) Kumasi, Ghana, in September 2018. The collected sample was authenticated by Mr. Clifford Osafo Asare, the horticulturist with the Department of Pharmacognosy, KNUST, and deposited in the Department's herbarium with a voucher specimen number of (003/10/07).

### 2.4. Isolation of Plumbagin

The powdered roots (2.4 kg) of *Plumbago zeylanica* were extracted with ethyl acetate for 5 days, and the extract obtained was concentrated with rotary evaporator (Buchi, Switzerland/R-114) at a temperature of 40°C and controlled vacuum pressure, to obtain a dried mass. Plumbagin (2.7 g) was eluted with a mobile phase composition of ethyl acetate (15% v/v) in petroleum ether (85% v/v) from a silica gel column.

### 2.5. Confirmation of Isolate

The isolate's identity was established with melting point, 1D-NMR (proton and carbon-13), and 2D-NMR (COSY, HMBC, HSQC, and DEPT-135) spectroscopy, with the support of infrared (IR) and ultraviolet-visible (UV-Vis) spectroscopy.

The melting point of the isolate was determined in triplicate with the Stuart melting point apparatus [UK/R000105350].

The UV-spectrophotometer (Jenway, UK/7315) was calibrated in the range of 200 to 700 nm of wavelength, with methanol as blank. A quantity (4 mg) of the isolate was dissolved completely in methanol and the UV-spectrum of the methanolic solution of the isolate subsequently determined.

The IR spectrum of the isolate was obtained using a Fourier-transform Infra-red (FTIR) (Bruker FTIR spectrometer) with the sample at a wavelength range of 4000 cm^−1^ to 400 cm^−1^.

Spectra for 1D-NMR (proton and carbon-13) as well as 2D-NMR techniques like COSY, HMBC, HSQC, and DEPT-135 were obtained with the Bruker Biospin NMR spectrometer (Billerica, US/F/NMR/A 175). The sample was run using deuterated chloroform (CDCl_3_) at a frequency of 500 MHz and temperature of 298 K.

### 2.6. Determination of Minimum Inhibitory Concentrations of Plumbagin and Standard Drugs

The minimum inhibitory concentration (MIC) of plumbagin and the reference drugs (ciprofloxacin, amoxicillin, ampicillin, and ketoconazole) were determined with the broth microdilution method. The 96-well microtitre plates were singly filled with 100 *μ*L of double strength nutrient broth and subsequently with different concentrations of plumbagin and reference drugs ranging from 0.25 to 256 *μ*g/mL. Twenty microliters of 10^6^ cfu/mL of test organisms were added to each well. Selected wells were filled with nutrient broth only and nutrient broth and organisms only, to serve as negative and positive controls, respectively. The microtitre plates were then incubated at 37°C for 24 h, after which 20 *μ*L of 1.25 mg/mL of 3-(4, 5-dimethylthiazol-2-yl)-2, 5-diphenyltetrazolium bromide (MTT) was introduced into each well and observations made for a purple coloration after 30 min of incubation, which signifies growth. The minimum concentrations of plumbagin and reference drugs that did not show any colour change in the wells were selected as the MIC. The experiment was carried out in triplicate [5].

### 2.7. Resistance Modulation Studies

The MICs of the antibiotics and antifungal were determined in the presence of subinhibitory concentration (4 *μ*g/mL) of plumbagin using the broth microdilution technique. The 96-well microtitre plates wells were singly filled with 100 μL of double-strength nutrient broth, appropriate volumes of different concentrations of the drugs, and 20 *μ*L of 10^6^ cfu/mL of test organisms. The plates were incubated for 24 h at 37°C, after which 20 *μ*L of MTT was introduced to the wells, and the MICs were determined as the lowest concentration at which no growth was observed by colour change to purple [5, 20–22].

### 2.8. Time-Kill Kinetics Assay of Plumbagin

Time kill kinetics of plumbagin was carried out using a modified procedure described by Appiah et al. [23]. The organisms employed (*Staphylococcus aureus* ATCC 25923, *Pseudomonas aeruginosa* ATCC 27853, *Escherichia coli* ATCC 25922, and *Candida albicans* ATCC 10231) were subcultured and diluted to 0.5 McFarland standard. Concentrations equal to MIC, twice the MIC, and four times the MIC of plumbagin were prepared and transferred into sterile broth in test tubes, after which an inoculum size of 1.0 × 10^6^ cfu/mL of the standardized organisms was added and the test tubes incubated for 37°C. Aliquots (1.0 mL) of the medium were taken at time intervals of 0, 6, 12, 18, 24, and 30 h for bacteria and 0, 6, 12, 30, 36, 48, 54, and 72 h for fungi and aseptically inoculated into nutrient agar in sterile Petri dishes. The agar was allowed to set, and the Petri dishes containing inoculum were incubated at 37°C for 24 h. A control test was performed alongside for the organisms only. The colony-forming unit (cfu) of the test organisms was determined and the procedure performed in triplicate. A graph of log CFU/mL was plotted against time. The data obtained from the study were analysed using one-way ANOVA followed by Dunnett's post hoc test from Graph Pad Prism Version 5.10 for windows (Graph Pad Software Inc., San Diego, CA, USA) [23].

### 2.9. Formation of Bacteria Biofilm

Pure cultures of the four bacteria (*Staphylococcus aureus* ATCC 25923, *Pseudomonas aeruginosa* ATCC 27853, *Klebsiella pneumoniae* ATCC 10031, and *Escherichia coli* ATCC 25922) diluted to 0.5 McFarland standard in Mueller–Hinton broth were employed. Ten microliters (10 *μ*L) of the standardized cultures was added to 100 *μ*L of broth in wells of a 96-well microtitre plate and the plate incubated at 37°C for 24 h. Planktonic cells were aspirated and the wells washed with sterile water to get rid of free floating bacteria after incubation. The bacteria biofilms formed in the wells were dried between 25°C to 28°C and stained with 0.1% crystal violet for 20 min. The stain was washed with sterile water and dried. The stained bacteria biofilm stuck to the wall of the wells were reconstituted in ethanol/acetic acid (1 : 1), and the absorbance read at 595 nm with a multimode microtitre plate reader. The optical density (OD) of the sterile broth was subtracted from that of the biofilm formed to eliminate background absorbance. The determination was performed in triplicate [24].

### 2.10. Inhibition Effect of Plumbagin on Biofilm Formation

Plumbagin and reference drug ciprofloxacin were reconstituted in dimethyl sulfoxide (DMSO) (1% v/v). Two-fold dilutions were made to achieve a concentration range of 1 to 128 *μ*g/mL. Aliquots of 100 *μ*L of double-strength Mueller–Hinton broth was added to the 96-well microtitre plate and the sample solution (plumbagin and ciprofloxacin) added. Ten microliters of microorganisms (24 h broth culture diluted to 0.5 McFarland standard and containing 10^6^ cells per mL) was added, and the plate was incubated at 37°C for 24 h. The planktonic cells were aspirated and the wells washed, dried, fixed, and stained after incubation. Absorbance was read at 595 nm and the optical density (OD) of the culture media control subtracted to obtain the inhibitory effects of plumbagin and ciprofloxacin. The determination was carried out in triplicate. The ability of the plumbagin and the reference drug (ciprofloxacin) to reduce the optical density (OD) compared to the negative control was considered the biofilm inhibitory activity [24]”

(1) $$\begin{array}{c} \%\;\mathrm{biofilm}\;\mathrm{inhibition}=\frac{\text{optical density}\left(\text{OD}\right)\text{ of control}-\text{OD of treatment}}{\text{optical density}\left(\text{OD}\right)\text{ of control}}\times 100. \end{array}$$

## 3. Results and Discussion

### 3.1. Characterization of Plumbagin

Plumbagin (1.5 g) was obtained (after recrystallization) as yellowish needle-like crystals with a melting point of 78–80°C. The retardation factor (*R*_f_) was obtained on a silica gel coated TLC plate developed with mobile phase of petroleum ether-ethyl acetate (70% : 30%) as 0.82.

The melting point of the isolated compound was obtained to be 78–80°C. This closely agrees with 78-79°C reported for plumbagin in the literature [13]. The sharp and undepressed nature of the melting point range obtained for the isolate emphasizes the purity of the compound.

**Figure 1:**
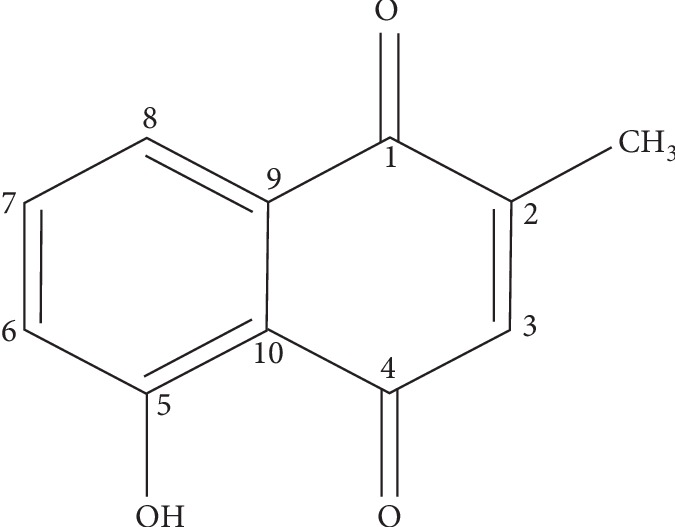
Chemical structure of plumbagin.

The UV-Vis spectrum of the isolate showed absorbance of 0.339 and 0.093 at wavelengths of 265 nm and 410 nm, respectively, indicating the presence of an extended conjugated chromophore system (Figure 1).

**Figure 2:**
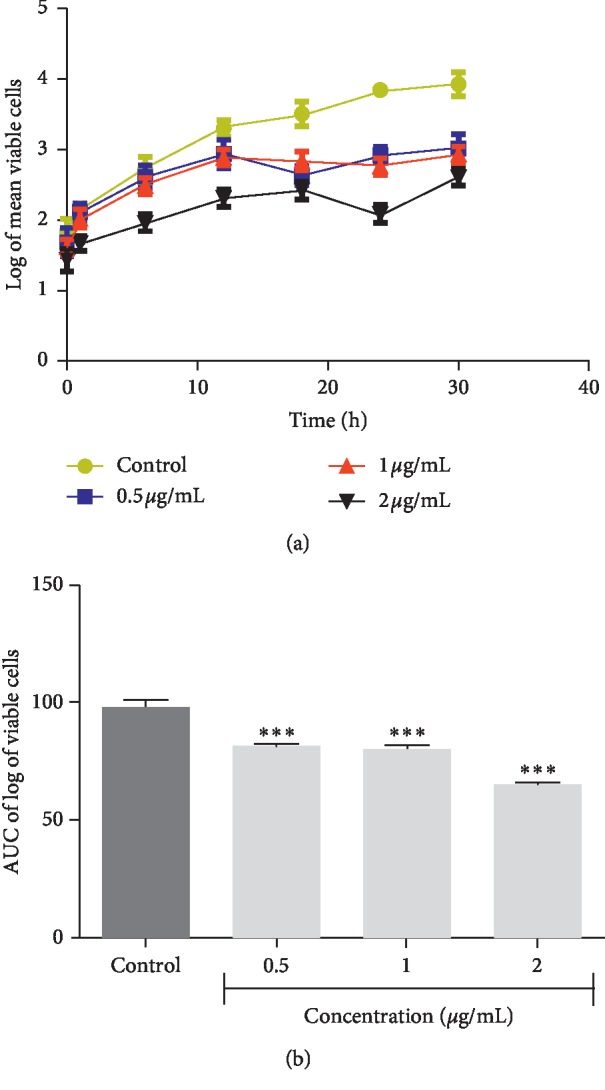
Time-kill kinetics of plumbagin against *Staphylococcus aureus*. (a) Time-kill kinetics curve and (b) AUC of time-kill kinetics. AUC: area under the curve. *n*=3; values are mean ± SEM. ^*∗∗∗*^*p* < 0.0001 (one-way ANOVA followed by Dunnett's post hoc test).

The IR spectrum showed a broad band at 3293 cm^−1^ that pointed to the OH group on the carbon at position 5. Peaks seen at wavenumbers 1161.60 cm^−1^ and 1639.66 cm^−1^ point to the free and hydrogen-bonded carbonyls at positions 1 and 4, respectively. Aromatic C=C vibrations observed at 1605.48 cm^−1^ is attributed to the carbons at positions 2 and 3, 5 and 10, 6 and 7, and 8 and 9. The absorption seen at 3038.4 cm^−1^ revealed –C-H stretches of aromatic Sp^2^ carbons (Figure 2).

The NMR data obtained further confirmed the isolated compound to be plumbagin. Chemical shifts and peak multiplicities obtained for the compound in the proton NMR analysis in deuterated chloroform (Table 1) bear similarities to the reported values in the literature [13]. The chemical shift integrals revealed a total of 8 protons which is in agreement with the molecular formulae of C_11_H_8_O_3_. The carbon-13 analysis of the compound in deuterated chloroform showed 11 carbons consistent with the molecular formula of plumbagin (Figure 1).

### 3.2. Minimum Inhibitory Concentration (MIC)

Growth was seen in all the positive controls which indicated that the nutrient broth sustained growth of the organisms employed. No growth was observed in the negative controls, implying that the equipment used and environment did not introduce microorganisms into the nutrient broth. The minimum inhibitory concentration (MIC) of plumbagin and the standard antimicrobials used against the test organisms is shown in Table 2.

### 3.3. Resistance Modulation Studies

The resistance modulation study involved determination of MICs of standard antimicrobials in the presence of subinhibitory concentrations of the agent(s) under investigation. Subinhibitory concentration is half the MIC of the agent being investigated for modulation effect that ordinarily does not inhibit the growth of microorganisms. MICs of the antimicrobials were redetermined at the concentration range of 0.25–256 *μ*g/mL in the presence of plumbagin's subinhibitory concentration of 4 *μ*g/mL chosen from a range of 0.25–4 *μ*g/mL at 37°C for 24 h. Decrease in MIC obtained implied potentiation of activities and increase in MIC suggested decrease in activities of the antimicrobial agents against the test organisms. Ciprofloxacin's activity was potentiated by 2-folds against *Staphylococcus aureus, Pseudomonas aeruginosa,* and *Escherichia coli*. The activity decreased by 2-folds against *Klebsiella pneumoniae*. Activity of amoxicillin was potentiated by 2-folds, 2-folds, and 6-folds against *Staphylococcus aureus, Escherichia coli,* and *Klebsiella pneumoniae*, respectively. The activity was however decreased by 2-folds against *Pseudomonas aeruginosa*. Ampicillin's activity was potentiated by 2-folds, 2-folds, and 6-folds against *Staphylococcus aureus, Escherichia coli,* and *Klebsiella pneumoniae*, respectively, and decreased by 2-folds against *Pseudomonas aeruginosa*. Ketoconazole's activity was potentiated by 12-folds against *Candida albicans* in the presence of subinhibitory concentration of plumbagin (Table 1).

### 3.4. Time-Kill Kinetics Assay of Plumbagin

**Figure 3:**
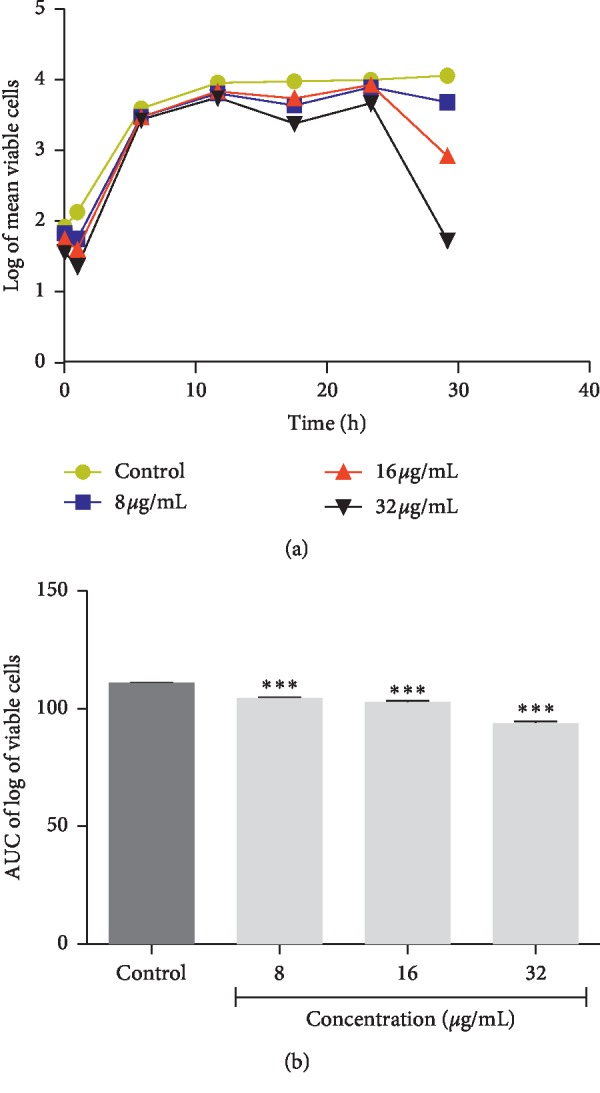
Time-kill kinetics of plumbagin against *Escherichia coli*. (a) Time-kill kinetics curve and (b) AUC of time-kill kinetics. AUC: area under the curve. *n*=3; values are mean ± SEM. ^*∗∗∗*^*p* < 0.0001 (one-way ANOVA followed by Dunnett's post hoc test).

**Figure 4:**
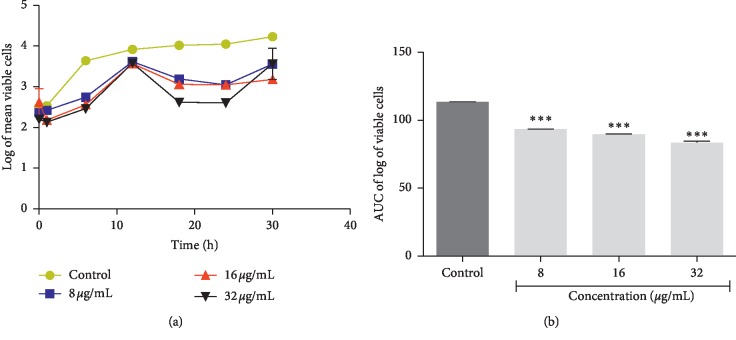
Time-kill kinetics of plumbagin against *Pseudomonas aeruginosa*. (a) Time-kill kinetics curve and (b) AUC of time-kill kinetics. AUC: area under the curve. *n*=3; values are mean ± SEM. ^*∗∗∗*^*p* < 0.0001 (one-way ANOVA followed by Dunnett's post hoc test).

**Figure 5:**
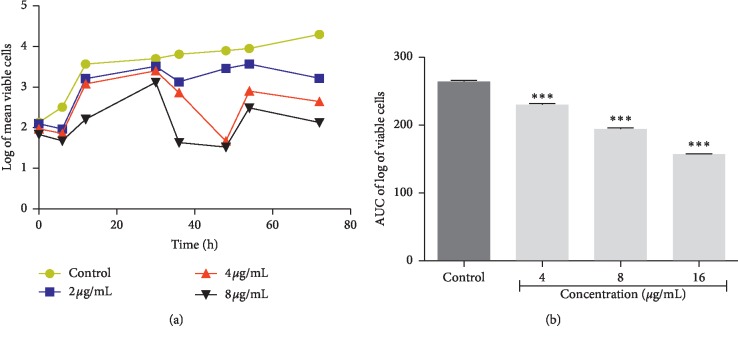
Time-kill kinetics of plumbagin against *Candida albicans*. (a) Time-kill kinetics curve and (b) AUC of time-kill kinetics. AUC: area under the curve. *n*=3; values are mean ± SEM. ^*∗∗∗*^*p* < 0.0001 (one-way ANOVA followed by Dunnett's post hoc test).

The time-kill kinetics profile of the isolated plumbagin against *Staphylococcus aureus* showed a reduction in the number of viable cells between 18 h and 24 h accompanied by an increase up to 30 hrs compared to the control (growth of organism without antimicrobial agents) (Figure 2). Time-kill kinetics profile of plumbagin against *Escherichia coli* at the test concentrations used showed a reduction in the number of viable cells between 12 h and 18 h, followed by a gradual rise up to the 24 hr and subsequent reduction up to the 30 h against its control (Figure 3). Plumbagin's time-kill kinetics profile against *Pseudomonas aeruginosa* at the various test concentrations showed a reduction in the number of viable cells between 12 h and 18 h, remained constant till 24 h, and followed by a gradual rise up to 30 h when compared to its control (Figure 4). Time-kill kinetics profile of plumbagin against *Candida albicans* at the test concentrations used showed a reduction in the number of viable cells between 30 h and 48 h, followed by a gradual rise up to 54 h and subsequent reduction to 72 h, compared to its control (Figure 5).

The antimicrobial effect of plumbagin was observed to be bacteriostatic against the test bacteria (*Staphylococcus aureus, Escherichia coli,* and *Klebsiella pneumoniae*) and fungistatic against the fungus (*Candida albicans*) used. The area under the curve (AUC) at the concentrations used showed significant reduction in the number of viable cells (*p* < 0.0001) compared to the negative controls (Figures 2–5).

### 3.5. Inhibition of Biofilm Formation Study

**Figure 6:**
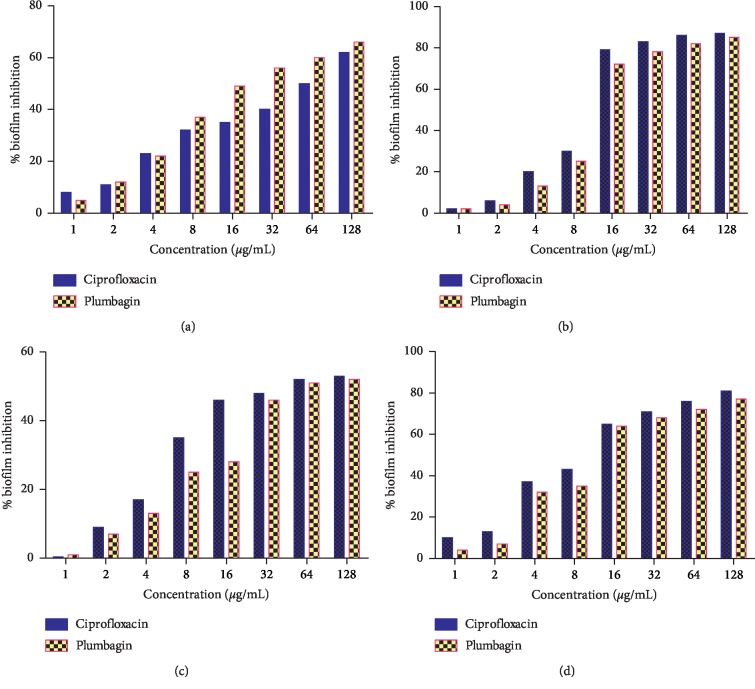
Percentage inhibition effect of plumbagin and ciprofloxacin (positive control) on biofilm formation by biofilm forming organisms: (a) *Staphylococcus aureus*, (b) *Escherichia coli*, (c) *Klebsiella pneumoniae*, and (d) *Pseudomonas aeruginosa*.

Plumbagin was assessed for its ability to either completely inhibit or reduce formation of bacteria biofilm using the concentration ranging from 1–128 *μ*g/mL. Ciprofloxacin was employed as positive control, and biofilm formed in the absence of antimicrobial agent was used as negative control. Plumbagin demonstrated maximum inhibition against *Escherichia coli* at 128 *μ*g/mL with 85% inhibition of biofilm formation, followed by 64 *μ*g/mL, 32 *μ*g/mL, and 16 *μ*g/mL with 82%, 78%, and 72% inhibitory effects, respectively. Inhibition against *Pseudomonas aeruginosa* biofilm formation was recorded for ciprofloxacin at 128 *μ*g/mL with 77% inhibition, followed by 64 *μ*g/mL, 32 *μ*g/mL, and 16 *μ*g/mL with 72%, 68%, and 64% inhibition, respectively. Plumbagin showed inhibition of *S. aureus* biofilm formation at 128 *μ*g/mL with 66% inhibition followed by 64 *μ*g/mL and 32 *μ*g/mL with 60% and 56%, inhibitions, respectively. Inhibition against *K. pneumoniae* biofilm formation was recorded for plumbagin at 128 *μ*g/mL with 52% inhibition followed by 64 *μ*g/mL with 51% inhibition (Figure 6).

Ciprofloxacin (positive control) showed maximum inhibition against *E. coli* biofilm formation at 128 *μ*g/mL with 87% inhibition, followed by 64 *μ*g/mL, 32 *μ*g/mL, and 16 *μ*g/mL with 86%, 83%, and 79% inhibition, respectively. Inhibition against *P. aeruginosa* biofilm formation was recorded at 128 *μ*g/mL with 81% inhibition, followed by 64 *μ*g/mL, 32 *μ*g/mL, and 16 *μ*g/mL with 76%, 71%, and 65% inhibitions, respectively. Inhibition against *S. aureus* biofilm formation was recorded at 128 *μ*g/mL with 62% inhibition followed by 64 *μ*g/mL with 50%. Inhibition against *K. pneumoniae* biofilm formation for ciprofloxacin was recorded at 128 *μ*g/mL with 53% followed by 64 *μ*g/mL with 52% inhibition (Figure 6).

## 4. Conclusion

Plumbagin (2.7 g) was successfully isolated from 2.4 kg of the powdered roots of *Plumbago zeylanica* by column chromatography of the ethyl acetate extract. Its identity was confirmed using physical properties and spectroscopic methods. Plumbagin demonstrated activity against *Staphylococcus aureus* ATCC 25923, *Escherichia coli* ATCC 25922*, Klebsiella pneumonia* ATCC 10031, *Pseudomonas aeruginosa* ATCC 27853, and *Candida albicans* ATCC 10231 with MICs of 0.5, 8, 2, 8, and 4 *μ*g/mL, respectively. Plumbagin successfully modulated activities of ciprofloxacin, amoxicillin, ampicillin, and ketoconazole, implying that it has the potential to be developed as a drug candidate for use alongside commercial antimicrobials in combination therapies, in drug discovery and development to combat the menace of antimicrobial resistance. Plumbagin has also been established through a time-kill kinetics study to be bacteriostatic and fungistatic. Plumbagin demonstrated strong biofilm inhibition at concentrations of 128, 64, and 32 *μ*g/mL, so as the reference drug ciprofloxacin, suggesting its potential in the fight against antimicrobial resistance known to result to a greater extent from biofilm formation by the panel of organisms involved.

## Figures and Tables

**Table 1:** MICs of standard antimicrobial drugs in the presence of subinhibitory concentration of plumbagin (4 *μ*g/mL).

Antimicrobials	MIC (*μ*g/mL)									
	*S. aureus*		*E. coli*		*K. pneumoniae*		*P. aeruginosa*		*C. albicans*	
	MIC	Folds	MIC	Folds	MIC	Folds	MIC	Folds	MIC	Folds
Ciprofloxacin	**0.5**	2 (+)	**0.25**	2 (+)	**2**	2 (−)	**0.25**	2 (+)		
Amoxicillin	**0.25**	2 (+)	**2**	2 (+)	**4**	6 (+)	**256**	2 (−)		
Ampicillin	**0.25**	2 (+)	**2**	2 (+)	**4**	6 (+)	**256**	2 (−)		
Ketoconazole									**4**	12 (+)

(+): reduction in MIC (increased activity). (−): increase in MIC (decreased activity).

**Table 2:** MICs of plumbagin and antimicrobials against test organisms.

Antimicrobials	MIC (*μ*g/mL)				
	*S. aureus*	*E. coli*	*K. pneumoniae*	*P. aeruginosa*	*C. albicans*
Plumbagin	**0.5**	**8**	**2**	**8**	**2**
Ciprofloxacin	**1**	**0.5**	**1**	**0.5**	
Amoxicillin	**0.5**	**4**	**32**	**128**	
Ampicillin	**0.5**	**4**	**32**	**128**	
Ketoconazole					**256**

## Data Availability

The data used to support the findings of this study are available at the Department of Pharmaceutical Chemistry, KNUST, Kumasi.
